# The reciprocal relationship between openness and creativity: from neurobiology to multicultural environments

**DOI:** 10.3389/fneur.2023.1235348

**Published:** 2023-10-11

**Authors:** Maison Abu Raya, Adedoyin O. Ogunyemi, Veronica Rojas Carstensen, Jake Broder, Maryenela Illanes-Manrique, Katherine P. Rankin

**Affiliations:** ^1^Global Brain Health Institute, University of California, San Francisco, San Francisco, CA, United States; ^2^Department of Neurology, Memory and Aging Center, Weill Institute for Neurosciences, San Francisco School of Medicine, University of California, San Francisco, San Francisco, CA, United States; ^3^Department of Community Health and Primary Care, University of Lagos, Lagos, Nigeria; ^4^Neurogenetics Research Center, Instituto Nacional de Ciencias Neurologicas, Lima, Peru

**Keywords:** openness to diversity, creativity, multicultural environment, salience network (SN), default mode network (DMN), executive control network (ECN)

## Abstract

The desire for novelty and variety in experiences, which may manifest in an inclination to engage with individuals from a diverse range of cultural backgrounds, collectively constitutes the personality dimension known as “Openness to Experience.” Empirical research has identified a positive correlation between trait openness and various expressions of creativity, such as divergent ideation, innovative problem-solving strategies, and cumulative creative accomplishments. This nexus between openness to interpersonal diversity, as an aspect of the larger personality trait of openness, and creativity has precipitated considerable scholarly interest across the disciplines of personality, social and organizational psychology, and neuroscientific investigation. In this paper, we review the neurobehavioral properties, including the cognitive processes and neural mechanisms, that connect these two constructs. Further, we explore how culture influences levels of openness and creativity in individuals and consider how creativity predisposes individuals toward openness to a plethora of experiences, including those occurring in culturally diverse contexts. This reciprocal entanglement of creativity and openness has been shown to foster a reduction in biases, augment conflict resolution capabilities, and generally yield superior outcomes in multicultural environments.

## Introduction

Openness, as a high-level construct within the Five-Factor Model of personality traits (1, 2), includes various facets such as imagination, perceptiveness, and intellect (3–5). These facets configure a spectrum of cognitive and behavioral patterns and habits (6) associated with various attributes such as broad-mindedness, creativity, intellectual sophistication, curiosity, cognitive flexibility, receptivity to diverse perspectives and cultural practices, desire for novelty, as well as appreciation for varied experiences, values, and beliefs (5, 7, 8).

Openness to experience, a key facet of this broader trait (4, 5), specifically pertains to the degree to which an individual is receptive to novel experiences and divergent forms of thought (5, 9). Individuals who score high on this trait tend to engage in a broad range of interests, are highly imaginative, and typically exhibit a heightened sensitivity to art and beauty. They are prone to introspection and often have an intricate and nuanced emotional life (4, 5, 7). Openness to experience can also specifically entail greater openness to diversity, meaning the predisposition to engage with, appreciate, and respect different perspectives and values, including those coming from other cultures (6). This dimension not only encapsulates a willingness to comprehend and accept cultural differences, but also to adopt new cultural practices and to challenge conventional norms when considering new perspectives and nontraditional values. An individual’s capacity for openness, particularly when expressed as an openness to diversity, influences their ability to navigate effectively and thrive in culturally heterogeneous environments, and has been shown to correlate with a reduction of in-group biases, improved intercultural communication, and better conflict resolution skills (6, 10–12).

Creativity, a distinct construct that is nonetheless highly related to the personality trait of openness, describes the mental agility needed to perceive and embrace novel esthetic and intellectual information in order to synthesize it with the goal of generating original ideas, concepts, and works of art (6, 9, 13, 14). While openness does not in itself presuppose generation of novel work but is limited to an attitude of receptiveness toward novelty, creativity by definition involves the production of novel intellectual, esthetic, or physical materials. Silvia and colleagues found that trait openness was a significant predictor of creative achievement across several domains, including writing, visual arts, and music (9). Individuals high in trait openness are more likely to engage in activities that expose them to a broad range of experiences, and this exposure can provide them with a greater repertoire of knowledge and ideas that can be drawn upon during the creative process (15, 16).

## Measuring the different facets of openness

The relationship between openness to experience and creativity is intricate, and they share interconnected measurement approaches within the psychological research domain. These measures serve as psychometric lenses, affording researchers nuanced insights into individuals’ cognitive and affective orientations toward novelty and diversity.

In psychometric parlance, openness as a personality trait is often assessed through various theoretically grounded behavioral scales that measure different facets by employing standardized self-reported or other-reported questionnaires. For instance, the NEO Personality Inventory is a widely used tool to measure openness. It assesses this trait across six facets: Fantasy, Esthetics, Feelings, Actions, Ideas, and Values (2, 10, 17). The Fantasy facet gauges a person’s level of imagination, creativity, and daydreaming tendency. Esthetics measures their appreciation of art, music, and beauty. Feelings examines emotional awareness, sensitivity, and intensity of emotions experienced. Actions evaluates adventurousness, risk-taking propensity, and preference for novelty. Ideas assesses intellectual curiosity, open-mindedness, and appreciation for new concepts. Lastly, the Values facet gauges openness to alternative belief systems, such as spiritual or religious beliefs (10). Additional questionnaire tools include the HEXACO model, which adds an emphasis on the ethical and moral aspects of openness while introducing the Honesty-Humility dimension (18, 19), and the California Psychological Inventory (CPI), which assesses traits that researchers believe are related to openness, such as intellectual efficiency, creativity, and esthetic appreciation (20).

However, openness is also measured via direct neuropsychological assessments where openness is conceived to be reflected by the volume and quality of creative output, conflating the constructs of openness and creativity. One such creativity measure is the Torrance Tests of Creative Thinking (TTCT), which evaluates the performance of tasks requiring divergent thinking and problem-solving skills, and gives examinees higher scores as a result of greater volume and novelty of output (21, 22).

In addition to measures examining openness more broadly, several other tools are used in research settings to explicitly quantify Openness to Diversity and Cultural Openness. The Multicultural Personality Questionnaire (MPQ) (23) assesses seven dimensions, including Cultural Empathy and Open-mindedness, which together gauge an individual’s openness to cultural diversity. The Intercultural Sensitivity Scale is another instrument measuring an individual’s ability to modify behavior in response to different cultural norms, thus capturing their level of cultural openness (24). The Miville-Guzman Universality-Diversity Scale (M-GUDS) serves as a measure of “universal-diverse orientation,” a psychological construct similar to openness to cultural diversity, which quantifies individuals’ comfort and interest in interactions with people from diverse cultural, racial, and social backgrounds (25).

## Shared cognitive processes and neural mechanisms underlying both openness and creativity

The cognitive processes underlying both openness to diversity and creative thinking involve divergent and convergent thinking, which are distinct but complementary cognitive processes (9, 14). Divergent thinking involves the ability to generate a wide range of possible solutions or ideas to a problem, often through brainstorming or free association. This involves exploring multiple perspectives and possibilities and is often associated with creativity and innovation. It is clear why divergent thinking supports openness to diversity, as it involves being open to a wide range of perspectives, experiences, and ideas from different cultural backgrounds. This can expand the range of possible solutions and approaches to a problem, leading to more creative and innovative outcomes. Openness to diversity can also facilitate divergent thinking by allowing one to make broader associations beyond any stereotypes or biases that may limit the range of ideas generated (14, 16).

However, divergent thinking alone may not lead to effective solutions or choices, as it can result in many possible but often unfeasible ideas. Convergent thinking involves the ability to narrow down a set of options to identify the most appropriate or effective solution to a problem. Yet, this activity is also supportive of both creativity and openness to diversity. This process underpins the drive to actively investigate available options with the goal of finding the most appropriate, often the novel solution in order to resolve a problem or conflict (3, 4, 9, 26), which is an essential component of effective creativity.

Another important promoter of both openness to diversity and creative thinking is the capacity to overcome biases, since implicit biases can act as a barrier to openness to novel cultures and other types of diversity. These biases are unconscious mental shortcuts and stereotypes that individuals use when processing information about others, which can lead to unfair judgments and discrimination against others, particularly when individuals are from cultures and backgrounds with which one has not had extensive experience. Biases can limit the range of perspectives and ideas generated, leading to narrow and uncreative solutions. By being mindful of their biases and actively challenging them, individuals can expand their range of perspectives and ideas, leading to more creative outcomes (14).

The neurocognitive processes underlying both creativity and the capacity for openness rely on an overlapping core of brain networks that include a large set of brain regions, including the amygdala, fusiform gyrus (FFG), insula, ventral striatum, locus coeruleus (LC), anterior cingulate cortex (ACC), posterior cingulate cortex (PCC), and lateral and medial regions of the prefrontal cortex (PFC) (16, 27–32). These structures work together in complex networks to help us perceive and interpret the world around us, consider new ideas and perspectives, draw on our own experiences and beliefs, inhibit biases and automatic responses, and appreciate the perspectives of others. As shown imaginatively in Illustration 1, these structures might be conceptualized as lenses and filters through which we receive and process information about the world, shaping the foundation from which we choose our beliefs and behaviors.

Initially, we perceive the world around us using different brain areas that receive different sensory inputs (visual, auditory, or tactile sensations) via the sensory cortex. An important brain region that is involved in visual perception in interpersonal contexts is the fusiform gyrus (FFG), which is involved in face recognition and therefore contributes to social cognition (29). The FFG is part of a larger network of secondary association cortex that includes the superior temporal sulcus (STS) and other regions that are important for recognizing and processing social information, such as emotional expressions, body language, and features relevant to social hierarchies and status.

Next, this sensory input automatically activates areas that are involved in our emotional reactions and our estimates of reward. Regions of the limbic system, like the amygdala, play a key role in processing emotional information, including fear and aggression, and the ventral striatum and other subcortical regions are involved in reward processing. These regions have also been long understood to directly underpin stereotyping and prejudice, as they contribute to both the negative emotions toward non-native ideas, individuals, and practices that engender exclusionary and antagonistic attitudes, as well as the positive appraisals and reward engendered by in-group individuals and behaviors (26, 29, 33, 34).

Once input passes through the sensory and the limbic systems, in the next stage, attentional and motivational processes engage and modulate the information, mediated by the insula and the dorsal anterior cingulate cortex (dACC), which together comprise the salience network (SN) (35–37). The anterior insula receives multimodal input comprised of a combination of sensory, affective, and visceral afferents, which it rapidly filters to determine what is relevant to the safety, survival, or well-being of the individual, and thus is worthy of additional attention. The dACC plays a role in motivating reactions to these multimodal inputs and modulating autonomic reactivity accordingly.

The SN interacts reciprocally with two other major networks: the default mode network (DMN), which uses internally generated experiences in decision-making, and the adaptive executive control network (ECN), which divides attention and exerts top-down control (16). More specifically, the DMN includes regions such as the medial prefrontal cortex (mPFC), posterior cingulate cortex (PCC), and inferior parietal lobule (IPL) (38). The DMN is the only internally-oriented network in the brain, in the sense that it does not directly respond to or act upon sensory stimuli, but engages in internally generated material such as memories, emotions, and predicted schemas about the world, and is thought to play a role in mind-wandering, introspection, creativity, and interpersonal perspective taking (35, 38). The ECN includes the dorsolateral prefrontal cortex (dlPFC) and the dorsolateral parietal cortex and is active during active thinking, planning, reasoning, and making decisions. While the DMN adds a self-referential, internal dimension to how our sensory and emotional inputs are processed, the ECN exerts top-down control of these inputs by adding the external dimension of conscious planning and reasoning prior to decision-making. The SN also contributes to these processes by monitoring the activity of the ECN and DMN and facilitating flexible switching of attention between the internal and the external streams of thought (26, 36). An important construct that modulates the interactions across those different networks is the noradrenergic system of locus coeruleus (LC) (36). The LC’s interactions with the limbic system, SN and DMN exert significant influence on visual and sensory processing by regulating the salience of stimuli, enhancing attention to novel or behaviorally relevant information, and modulating sensory gain, which extends to shaping biased behaviors, attitudes, and responses to novelty (27, 28, 36).

The contribution of each of these brain networks to both openness and creative cognition is summarized in Tables 1, 2.

**Table 1:** Neural regions and networks associated with openness and creativity.

Brain region	Role in openness and processing of stereotypes	Role in creative processing
**Limbic and salience networks**		
Ventral Striatum/vmPFC	Involved in reward processing, reinforcement learning, and emotion regulation, which can influence one’s openness to diversity and motivation to seek out new experiences and perspectives (39, 40).	Play a role in creative cognition by integrating emotional and motivational factors into idea generation and evaluation. Of potential outcomes of different decisions and choose the most innovative and rewarding option (41).
Amygdala	Provides automatic alerting signals in response to previously learned threats and rewards. This contributes to generating emotional responses associated with stereotypes and biases, which may influence the degree of openness to previously threatening experiences or ideas (29, 33, 42, 43).	Alerts to stimuli that are novel or have emotional valence, allowing attention to novel and stimulating ideas and experiences (29).
Insula	Involved in emotion processing and interoceptive awareness, which can influence one’s attention to different experiences and perspectives (42).	Plays a role in the creative process by integrating emotional and bodily signals to guide idea generation and evaluation (44).
dACC	Involved in conflict monitoring and error detection, which can facilitate perspective-taking and overcoming biases in social contexts (29, 43, 45).	Plays a role in the degree to which one is likely to reinterpret novel, complex, or conflicting information as aversive vs. positive. Involved in behavioral motivation, thus can influence the degree to which one seeks out novel experiences and ideas (26, 31).
FFG and other cortical secondary association areas	Involved in the higher-order processing and interpretation of sensory information, including faces, voice prosody, and body postures and gestures, which can influence one’s perception of and attitudes toward diversity (29).	May contribute to creativity by facilitating the recognition and association of novel visual or other sensory stimuli in a socio-emotional context (31, 46).
**The default mode network**		
dmPFC	Involved in aspects of social cognition that rely on perspective-taking and self-referential thought, such as mentalizing about similar and dissimilar others, which is crucial for developing intercultural sensitivity and overcoming stereotypes (47).	Self-awareness, mentalizing, and autobiographical memory allow us to connect emotionally with others as well as draw upon our own experiences and perspectives to generate new and innovative ideas (48).
	Also maintains a schema for predictions and expectations in a social context on the basis of autobiographical experience, which can influence one’s cognitive biases and expectations in diverse or novel situations (29, 49).	Plays a role in creative cognition by facilitating the integration of multiple sources of information and experience for predictions and concept generation, supporting the generation of novel solutions and spontaneous ideas (44, 49).
PCC	Involved in self-referential processing and perspective-taking, allows us to see things from different points of view and consider alternative perspectives and be more open-minded when evaluating new ideas (29).	May be involved in divergent thinking and the processing of creative stimuli (26, 44).
IPL	Involved in sensory integration and spatial cognition, which can support perspective-taking and cognitive flexibility (29).	May play a role in the creative process by facilitating the manipulation and transformation of mental representations (44).
**The executive control network**		
IFG	Involved in inhibiting automatic responses and generating “stop signals,” as well as engaging reappraisal of emotions by relabeling emotional experiences, and thus contributes to regulation and moderation of automatic stereotypes and biases (29).	Supports creativity through its function in detecting novelty and redirecting attentional resources toward novel stimuli (31, 50).
dlPFC	Exerts top-down cognitive control, set-shifting, inhibitory processes, and goal-directed behavior, which can support cognitive flexibility and openness to different viewpoints. This contributes to the explicit inhibition of biased thoughts and behaviors and the promotion of flexible and nuanced representations of others (29, 43).	Plays a role in the creative process by facilitating idea generation, evaluation, and selection (44, 50).

vmPFC, Ventromedial Prefrontal Cortex; dACC, Dorsal Anterior Cingulate Cortex; PCC, Posterior Cingulate Cortex; FFG, Fusiform Gyrus; IPL, Inferior Parietal Cortex; IFG, Inferior frontal gyrus; dmPFC, Dorsomedial Prefrontal Cortex; dlPFC, Dorsolateral Prefrontal Cortex.

**Table 2:** Summary of recent studies on openness, bias processing, and creativity.

Study	Sample	Creativity task/questionnaires	Methodology and imaging analysis	Results summary and interpretation
Wei et al. (2014)	*N* = 269 Healthy individuals	Divergent thinking measured by the Torrance Tests of Creative Thinking (TTCT).	Pre and post-task –resting state fMRI Whole-brain voxel-based activity and ROI-functional connectivity	Study findings suggest that increased RSFC between the default mode network’s mPFC and mTG, may be essential for creativity and that cognitive stimulation can increase RSFC between these two brain regions (reflecting creativity training-induced changes in functional connectivity, especially in the lower creativity individuals who had lower scores of Torrance Tests of Creative Thinking) (49).
Beaty et al. (2020)	*N* = 23 Healthy individuals	(EI) and semantic (SI) induction tasks, Alternate uses task AUT	fMRI Multivoxel patterns of neural activity	In comparison to episodic induction, semantic induction and subsequent generation were characterized by greater pattern similarity within the left AG, left IPL, and PCC, suggesting that these regions contributed to semantic processing throughout the AUT (51).
Li et al. (2016)	*N* = 304, healthy individuals	TTCT-F, CRT	fMRI Seed-based functional connectivity	Results showed a correlation between higher creativity and reduced RSFC between the mPFC and precuneus and increased RSFC between the left and right dlPFC (46).
Beaty et al. (2018)	*N* = 163 Healthy adults.	Creative ideation task, Alternate uses task (AUT) of divergent thinking	Resting-state and task-based fMRI Two task-based fMRI samples and one task-free resting-state sample fMRI during creative ideation task Functional connectivity analysis	Greater default mode network, SN, and ECN functional connectivity is associated with higher creativity and divergent thinking (44).
Li et al. (2015)	*N* = 246	Raven’s Advanced Progressive Matrix (RAPM), Raven’s Advanced Progressive Matrix (RAPM, WCAT) (The Creativity Assessment Packet.)	Structural volumetric MRI	These findings suggest that an individual’s trait creativity may be significantly influenced by the specific personality trait of openness to experience and that creativity and the appropriate pMTG volume are related through openness to experience to some extent (16).
Marstrand-Joergensen et al. (2021)	*N* = 295	Openness to experience (NEO-PI-R)	Resting-state fMRI Functional connectivity	Resting state connectivity within the DMN was negatively associated with trait openness, including the Fantasy aspect (52).
Wang et al. (2022)	*N* = 39 Healthy individuals	The Creative Achievement Questionnaire (CAQ), Creative Behavior Inventory (CBI), The Biographical Inventory of Creative Behaviors (BICB) To assess divergent thinking: the Product Improvement Task (PIT), The Alternate Uses Task (AUT). Openness to experience (NEO-PI-R)	fMRI functional connectivity	At the behavioral level, there is a correlation between creative achievement and both experiential openness and diverse thinking. Both openness to new experiences and diverse thinking involves the attentat networks and the default mode network since they both call for focus and the capacity for spontaneous thought (53).
Sun et al. (2019)	*N* = 29 healthy adults	Divergent thinking task AUT, control task (OCT-object characteristic task), NEO-Personality Inventory	Resting-state fMRI Activation analysis	Different combinations of network connectivity patterns predict creativity and openness to experience. The results showed that the precuneus and middle temporal gyrus were positively related to the inferior parietal lobule. Positive connections between the precuneus and supramarginal gyrus and the middle frontal gyrus/superior frontal gyrus were found. Individual difference analysis showed a significant correlation between openness to experience and the intensity of FCs between various important default mode, cognitive control, and salience network areas. The network-based mechanisms that underlie creativity and the neurological foundation of individual differences in openness to experience were found to be true (13)
Firat et al. (2017)	*N* = 17 patients with focal brain lesions (vmPFC or amygdala)	The International Affective Picture System (IAPS), the World Wide Web	fMRI Activation analysis	Higher activation of the amygdala in situations individuals had to assess out of group race. These findings show that the amygdala may be encoding other socially valued face characteristics in addition to automatically classifying people into various ethnic groupings. The results suggested a probable involvement of different brain areas in class-based racial assessments: the amygdala for the lower and upper classes and the vmPFC for the middle class (40).
Sakaki et al. (2020)	*N* = 40 Healthy Japanese University Students	Cognitive bias modification for interpretation (CBM-I) tasks, vs. a control group who received positive and negative ending written scenarios. For the assessment test, only the first two sentences were displayed for a total of 10 s. The first two sentences were left up to the participants’ interpretation, and they were to think of a possible outcome for the scenario and envision it for 10 s.	Task-based fMRI	Participants perceived novel social scenarios. Whole-brain analysis revealed group-self-awareness interaction, altering brain activity in various areas, including the somatomotor and somatosensory areas, occipital lobe, and Post. Cingulate Gyrus, right amygdala, The hypothesis that the individuals’ imagery was altered to be processed as higher social reward may also be supported by the increase in visual cortex activity and reinforced functional connections between the ACC and DLPFC that coincided with SA reduction. Interaction between areas of memory retrieval were also shown by high functional connectivity between IPL PCG, and SFG interact with the ACC, possibly indicates participants’ attempts to retrieve and recall positive interpretations for ambiguous social circumstances in a self-referential manner (29).

In curating articles for our review on the relationship between openness and creativity neurobiology, we employed a methodical approach, prioritizing studies with rigorous neuroscientific methodologies, particularly those involving empirical research and neuroimaging techniques.

## The reciprocal relationship between openness to cultural diversity and creativity

Several studies suggest a reciprocal relationship in which openness to experience more generally, and openness to cultural diversity more specifically, act to foster creativity by generating greater exposure to new and diverse cross-cultural interactions (9, 15). Multicultural exposure, when facilitated by a habit of openness, encourages individuals to be curious about and investigate novel perspectives, ideas, and beliefs, even when they are different from their own. The process of gaining a deeper understanding of the nuances that make each culture unique provides new knowledge and inspiration for innovative thinking and problem-solving (54). Subsequently, individuals are thereby more likely to creatively incorporate various novel cultural influences into their work, which facilitates cognitive flexibility and the generation of new ideas in a positive feedback loop (15, 55). Moreover, exposure to diverse cultural perspectives has been demonstrated to help individuals to better understand and empathize with others, which in turn facilitates interpersonal conflict resolution (16, 18).

Reciprocally, creativity leads to greater cultural openness by challenging existing norms and encouraging the exploration of new ideas as a means to resolving conflicts (19, 56). Creativity has the potential to bridge cultural differences and facilitate conflict resolution by generating novel and innovative solutions that are sensitive to diverse cultural perspectives. This may be particularly relevant in situations where traditional approaches have failed. They suggest that a culturally sensitive approach to creativity can promote intercultural understanding and dialog, leading to more effective conflict resolution in multicultural environments (57–59).

## The interplay of diversity and creativity in real-world multicultural environments

Globalization has accelerated, and there have been an increasing number of studies examining how cultural differences are managed in light of the internationalization of organizations and, in particular, the rising number of staff members from various cultures. Attitudes toward individualism and conformity changed in the 1980s, at both societal and organizational levels, and this shift has been attributed to several factors that include the impact of multiculturalism and interpersonality (60). Multiculturalism refers to the recognition and celebration of diversity within society, while interpersonality refers to the importance of interpersonal relationships and connections (61–63).

Research by Amabile et al. found that exposure to multiculturalism increased creativity and innovation, as individuals were exposed to new ideas and perspectives. They hypothesized that this, in turn, may have contributed to the growing emphasis on individualism in the 1980s, as individuals were encouraged to pursue their own unique interests and goals in order to boost the success of organizations. Similarly, the importance of interpersonality in the 1980s may have contributed to the shift in attitudes away from conformity. As individuals formed stronger interpersonal connections, they may have felt less pressure to conform to societal norms and expectations, and more empowered to pursue their own goals and interests (60, 64).

In the late 1980s, the theoretical concept of “organizational creativity” was first suggested by Woodmann, who put the term “creativity” in the organizational context in reference to the creative process of individuals who work together in a complex social setting for creating an innovational and useful product (65). One commonly cited model of organizational creativity that has often been used in organizational creativity studies is the Five-Stage Model, developed by Teresa Amabile and colleagues (66, 67). The model includes five stages: Problem Identification, Preparation, Idea Generation, Idea Evaluation, and Outcome Assessment (66, 67). It has been noted that while the first three stages are held in common between individual-level and organizational-level innovation, the remaining two are uniquely related to organizational environment factors such as resources, material systems, and general atmosphere, which may fortify or inhibit creativity in the organizational culture (68). Furthermore, studies have found that organizational diversity was positively related to the generation of new ideas when employees were highly engaged in the creative process. This suggests that organizations must create a supportive environment that encourages employees to engage in the creative process in order to fully benefit from the diversity of their workforce (64, 66).

Studying the collectivistic and individualistic approaches to organizational culture has provided insights into how individual creativity and organizational innovation may interact with each other. The collectivistic approach promotes conformity in a diverse group in service of a common goal, values, or mutual interest, avoiding individualistic values in order to minimize conflicts and opportunism (69). Studies have shown that when a collectivistic orientation is maintained in a diverse organizational culture that shares the common goal of productive, innovative work outcomes, it increases harmony, cooperation, a sense of identification with the work ingroup, and group cohesion (70, 71).

On the other hand, the individualistic approach is more likely to foster creativity on an individual level and is generally associated with a Western cultural mindset about the workspace. Organizational research has indicated that creativity is often an outcome of individuals deviating from consensual normative practices rather than maintaining them, as is more common in collectivistic work cultures (72). Recent research has shown that in order to promote “organizational creativity” in a complex social environment, individualistic values are beneficial, especially when the desired outcome of the work is a creative product (73). Social psychology studies have suggested that another advantage of the individualistic approach is that in a multicultural setting where individuals are accepting of diverse values and perspectives, facilitation of creative performance occurs that is mediated by an increased generation of uncommon and unconventional ideas, as well as by that culture’s enhanced receptiveness to ideas that are rooted in non-native cultures (15). Studies have shown that individuals who have experience living in a culture different from their own, and who needed to adapt to that non-native culture, have higher levels of creativity (54).

Additionally, recent studies provide evidence that the likelihood that cultural openness will positively impact creativity and conflict resolution in multicultural environments is mediated by the degree to which the multicultural team feels a sense of psychological safety and inclusiveness (14, 57). Thus, a culturally sensitive approach to creativity can promote intercultural understanding and dialog, fostering a safe and inclusive multicultural environment that facilitates conflict resolution and fosters individuals’ creative contributions. Reciprocally, creative thinking helps to generate new and innovative solutions to interpersonal disputes, particularly by seeking resolutions that consider different perspectives and satisfy the needs and interests of all parties involved by going beyond traditional, existing options. The use of creative techniques can help break down communication barriers, enhance collaboration, and promote understanding among conflicting parties as well. This creates a beneficial cycle leading to more positive intergroup attitudes and behaviors (5, 37, 50, 51).

Finding the best approaches to train teams to be more creative has also been a topic of recent investigation (58, 59). Effective training models suggest that it is possible to get positive effects out of diverse teams by building and preparing them systematically for team-driven creative tasks, supporting and preparing them not only cognitively but also motivationally, emotionally, and environmentally to contribute to the teams’ creative output (55, 58, 74).

## Conclusion

Openness to culturally diverse individuals and ideas is an important behavioral trait that is increasingly necessary for success in today’s interconnected world. By promoting inclusivity, individuals can create more diverse and innovative networks that foster creativity, collaboration, and growth in groups and organizations. Implicit biases can act as barriers to openness and inclusivity and reduce the organization’s creative output and effective problem solving; thus, it is necessary for individuals and organizations to identify and overcome such biases. Research consistently points to a largely shared neurobiology between creativity and trait openness, where common brain networks (i.e., the reward system, DMN, and ECN) facilitate divergent, convergent, and associative thought processes that play an important role in generating new critical perspectives and getting beyond automatic stereotypes to make further creative associations. While openness to diversity is one aspect of the larger trait of openness, more research with harmonized methodology is needed to directly and explicitly examine the relationship between the two, and to identify factors that may inhibit intercultural openness even in individuals who otherwise show high levels of trait openness. Further research is also needed to clarify the intricate dynamics between openness as a personality trait and the cognitive abilities that comprise it.

In the new era of globalization and multicultural organizational environments, creativity and openness to diversity are important tools when training multicultural groups to collaboratively solve conflicts. The evidence reveals that creativity can help resolve conflicts via the generation of new and innovative solutions, but it also shows that a reciprocal relationship exists in which openness to diversity and multicultural appreciation can enhance group creativity. This highlights the value of including and encouraging a diverse array of individuals within a collective. Supporting “organizational multiculturism” creates an environment that embraces and motivates divergent ideas and diverse individualistic values and creates safety for unconstrained creativity and freedom of thought. Promoting both openness to diversity and creativity can have a significant positive impact on individuals, organizations, and societies as they seek to implement effective solutions and resolve conflicts.

## Author contributions

The manuscript benefited from the collective input of all authors during the conceptualization stage having all authors taking part in developing the ideas for this manuscript. KR and MAR played a significant role in designing and structuring the paper. The initial draft was written by MAR, who served as the first author, while AO, VR, JB, MI-M, and KR contributed by reviewing and making edits. In addition, VR contributed to the visual representation of a creative illustration depicting various concepts related to neuroanatomy of creativity and openness. KR supervised the work and contributed to the writing, reviewing, and editing processes. All authors contributed to the article and approved the submitted version.

## Supplementary material

ILLUSTRATION 1 An illustrated creative analogy that incorporates the different brain regions and associated networks involved in both openness and creativity, by Veronica Rojas Carstensen, Visual artist, Atlantic Fellow for Brain Health Equity, GBHI, UCSF. This imaginative illustration shows the brain as a complex camera with different lenses and filters that represent the different brain areas involved in creativity and processing of stereotypes and biases. These regions and networks act as lenses and filters that perceive, shape, and project our interpretations and attitudes toward the world around us and act accordingly. dlPFC, Dorsolateral Prefrontal Cortex; ACC, Anterior Cingulate Cortex; mPFC, Medial Prefrontal Cortex; PCC, Posterior Cingulate Cortex; IFG, Inferior frontal gyrus.
