# Supplementary figures and images for: The reciprocal relationship between openness and creativity: from neurobiology to multicultural environments
Source: Front Neurol. 2023 Oct 11;14:1235348. doi: 10.3389/fneur.2023.1235348 (PMC10598598; doi:10.3389/fneur.2023.1235348)

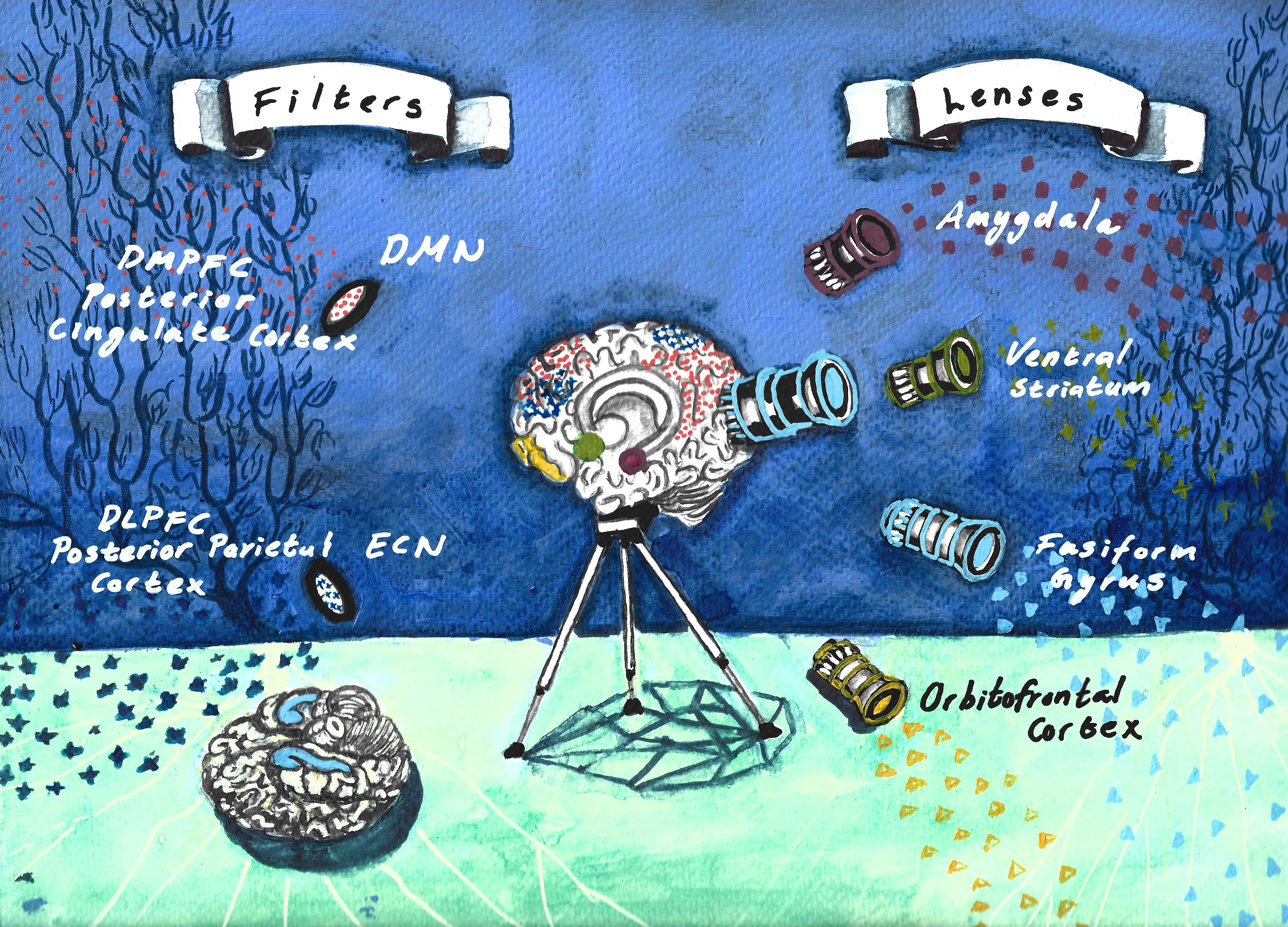

Supplement: ILLUSTRATION 1 — An illustrated creative analogy that incorporates the different brain regions and associated networks involved in both openness and creativity, by Veronica Rojas Carstensen, Visual artist, Atlantic Fellow for Brain Health Equity, GBHI, UCSF. This imaginative illustration shows the brain as a complex camera with different lenses and filters that represent the different brain areas involved in creativity and processing of stereotypes and biases. These regions and networks act as lenses and filters that perceive, shape, and project our interpretations and attitudes toward the world around us and act accordingly. dlPFC, Dorsolateral Prefrontal Cortex; ACC, Anterior Cingulate Cortex; mPFC, Medial Prefrontal Cortex; PCC, Posterior Cingulate Cortex; IFG, Inferior frontal gyrus. [file Image_1.jpg]
